# Hepatectomy After Progressive Disease Following Chemotherapy for Colorectal Liver Metastases: A Japanese Nationwide Cohort Study

**DOI:** 10.1245/s10434-026-20283-2

**Published:** 2026-08-01

**Authors:** Kazuki Kobayashi, Yoji Kishi, Naoto Yonamine, Takazumi Tsunenari, Mikiya Takao, Takahiro Einama, Hironori Tsujimoto, Hideki Ueno, Hirotoshi Kobayashi, Toru Beppu, Megumi Ishiguro, Kiyoshi Hasegawa, Kazushige Kawai

**Affiliations:** 1 Department of Surgery, National Defense Medical College, Tokorozawa, Saitama 359-8513 Japan; 2 Department of Surgery, Teikyo University Hospital, Mizonokuchi, Tokyo, Japan; 3 Department of Surgery, Yamaga City Medical Center, Kumamoto, Japan; 4 Health Science Research & Development Center, Institute of Science Tokyo Hospital, Tokyo, Japan; 5 Department of Surgery, Graduate School of Medicine, Hepato-Biliary-Pancreatic Surgery Division, The University of Tokyo, Tokyo, Japan; 6 Department of Colorectal surgery, Tokyo Metropolitan Cancer and Infectious Diseases Center Komagome Hospital, Tokyo, Japan

**Keywords:** Chemotherapy, Colorectal liver metastasis, Hepatectomy, Metachronous colorectal liver metastasis, Progressive disease

## Abstract

**Purpose:**

We evaluated the postoperative outcomes of patients with progressive disease after systemic chemotherapy compared with those with controlled disease.

**Methods:**

This retrospective study analyzed patients from the Colorectal Cancer Liver Metastasis Database, compiled by the Joint Committee of Liver Metastases Survey from Colorectal Cancer. Patients who received chemotherapy were categorized into complete response, partial response, stable disease, and progressive disease groups and further subdivided into resected and non-resected subgroups. Survival outcomes and prognostic factors were evaluated.

**Results:**

The number of patients in the complete response/partial response, stable disease, and progressive disease groups was 867, 550, and 195, respectively. Among the progressive disease group, 111 patients (57%) underwent hepatectomy. Their postoperative 5 year overall survival rate was 42.7%, which was significantly lower than that of the resected-complete response/partial response (62.0%, *p*<0.001) and resected-stable disease groups (55.5%; *p*<0.001). However, overall survival from the date of colorectal liver metastases diagnosis of the resected-progressive disease group was better than that of the non-resected-partial response group (5 year, 46.5% vs 11.4%, *p*<0.001). Multivariate analysis performed on the resected-progressive disease group revealed preoperative carcinoembryonic antigen (CEA) ≥100 ng/mL and metachronous colorectal liver metastases as independent predictors of poor overall survival.

**Conclusions:**

Although progressive disease after chemotherapy generally indicated poor prognosis, it should not be an absolute contraindication for hepatectomy. Preoperative CEA ≥100 ng/mL and metachronous colorectal liver metastases may identify a higher-risk subset and should be considered when determining surgical indications.

**Supplementary Information:**

The online version contains supplementary material available at https://doi.org/10.1245/s10434-026-20283-2.

Liver is the most frequent site of distant metastasis in colorectal cancer and represents a significant clinical challenge in terms of disease management and treatment optimisation.^1,2^ Surgical resection remains the mainstay of curative treatment for colorectal liver metastases (CRLM)^3–5^; however, its feasibility depends on factors such as tumor number and size, liver function, and the presence of extrahepatic metastases. When these factors preclude surgery, systemic chemotherapy is frequently used as the initial treatment approach.^6^ Response to chemotherapy is usually defined according to the Response Evaluation Criteria in Solid Tumors (RECIST) criteria,^7^ which categorize outcomes into complete response (CR), partial response (PR), stable disease (SD), or progressive disease (PD).

Viganò et al.^8^ reported that carcinoembryonic antigen (CEA) ≥200 ng/mL, CRLM number >3, and tumor size ≥50 mm were independent prognostic factors among the patients with PD following preoperative chemotherapy, and 5 year overall survival (OS) of those without any of these risk factors was 53.3%, concluding that PD was a poor prognostic factor but not an absolute contraindication of hepatectomy. However, their study focused on patients who underwent hepatectomy between 1990 and 2009; since then, chemotherapy regimens for CRLM have changed with the advent of molecular-targeted agents.^9,10^ Therefore, it would be meaningful to evaluate the prognosis and prognostic factors of patients with PD after chemotherapy in the more recent era.

We evaluated the postoperative outcomes of patients with PD after systemic chemotherapy compared with those with controlled disease (CR, PR, or SD). Additionally, factors associated with poor postoperative outcomes in patients with PD were assessed.

## Methods

### Patients and Study design

The patients in this study comprised those registered in the Joint Committee of Liver Metastases Survey from Colorectal Cancer between 2005 and 2017, with data from 2008 to 2012 not collected and, therefore, excluded from the database. This database included 11,647 cases collected from 209 departments at 201 institutions. All data were registered using the original comprehensive database system created by the Joint Committee via linkable anonymous codes that could only be connected and used within each facility before registration, thereby protecting personal information.

From the database, selected patients had undergone curative (R0) surgical resection of the primary colorectal cancer and their initial treatment for CRLM was systemic chemotherapy. Patients who received primary colorectal resection following chemotherapy for synchronous liver metastases were also included. The exclusion criteria were: (i) pathological diagnosis of endocrine cell carcinoma, (ii) extrahepatic metastases other than hepatic hilar lymph nodes at CRLM diagnosis, (iii) macroscopically non-curative (R2) hepatectomy, (iv) treated with radiofrequency ablation in combination with hepatectomy, and (v) incomplete clinical data.

The treatment efficacy of CRLM was assessed based on RECIST version 1.1,^7^ and all patients for the analyses were categorized into three groups as follows: CR/PR, SD, and PD groups. Patient demographics, primary tumor status, CRLM status, and chemotherapy regimen were assessed across all three groups. The patients in each response group were further divided into those who underwent hepatic resection (R-) and those who did not (nR-). The indication for hepatectomy after chemotherapy and the resection strategy of the lesions with CR were determined at the discretion of each participating institution. Disease-free survival (DFS) and cumulative incidence of recurrence were compared between the three groups with resection (R-CR/PR, R-SD, and R-PD), and OS was compared among the six groups (R-CR/PR, R-SD, R-PD, nR-CR/PR, nR-SD, and nR-PD). Univariate and multivariate analyses using Cox proportional hazards model were performed on the R-PD group to identify independent prognostic factors.

### Statistical Analysis

Continuous variables are expressed as medians and interquartile ranges. Statistical comparisons of baseline data were performed using the Mann–Whitney U-test, χ^2^ test, or Fisher’s exact test. Kaplan–Meier curves and log-rank tests were used to compare OS and DFS. The cumulative incidence of recurrence at 6 months and 1 year was estimated using the cumulative incidence function based on the Aalen–Johansen estimator, considering death without recurrence as a competing event. Group differences were assessed using Gray’s test. The Cox proportional hazards model was used to perform univariate and multivariate OS analyses. To assess the robustness of our findings to potential unmeasured confounding, we calculated E-values for the hazard ratios obtained from the multivariable Cox regression analyses.^11^ All statistical analyses were performed using EZR (version 1.68, Saitama Medical Center, Jichi Medical University, Saitama, Japan), which is a graphical user interface for R (version 4.20, The R Foundation for Statistical Computing, Vienna, Austria) and, more precisely, a modified version of R commander designed to add the statistical functions frequently used in biostatistics. Statistical significance was defined as p<0.05. For multiple pairwise comparisons of DFS and OS among groups, p-values were adjusted using the Bonferroni correction.

### Definitions

The therapeutic response to chemotherapy was assessed prior to hepatic resection in the patients with surgical resection when systemic chemotherapy was ceased and other non-chemotherapy treatment including best supportive care was planned.^7^ PD was defined according to RECIST version 1.1 as at least a 20% increase in the sum of the diameters of the target lesions, taking the smallest sum recorded since treatment initiation as reference, with an absolute increase of at least 5 mm. The appearance of one or more new lesions was also considered progression.

Pathologic stages were assessed following the TNM classification system of the Japanese Classification of Colorectal Carcinoma, 9^th^ edition.^12^ The cutoff values for tumor number, tumor size, and tumor markers were defined based on previous studies as five or more tumors,^13–15^ largest tumor diameter >50 mm, and CEA ≥100 ng/mL, respectively. DFS was defined as the time from initial liver resection to recurrence or death, and the patients alive without cancer recurrence were censored at the last follow-up date. OS was calculated from the date of CRLM diagnosis. For the analyses limited to the patients who underwent hepatectomy, OS was also assessed from the date of hepatectomy. To estimate the OS, patients who remained alive were censored at the date of the last follow-up. Synchronous CRLM was defined as the presence of hepatic metastases recognized before or at surgical exploration of the primary malignancy. Local liver recurrence was defined as tumor recurrence adjacent to the previous liver transection plane. Distant liver recurrence was defined as tumor recurrence within the remnant liver, away from the previous liver transection plane. Extrahepatic recurrence was defined as distant metastasis to other organs outside the liver.

## Results

### Patient Selection

**Fig. 1:**
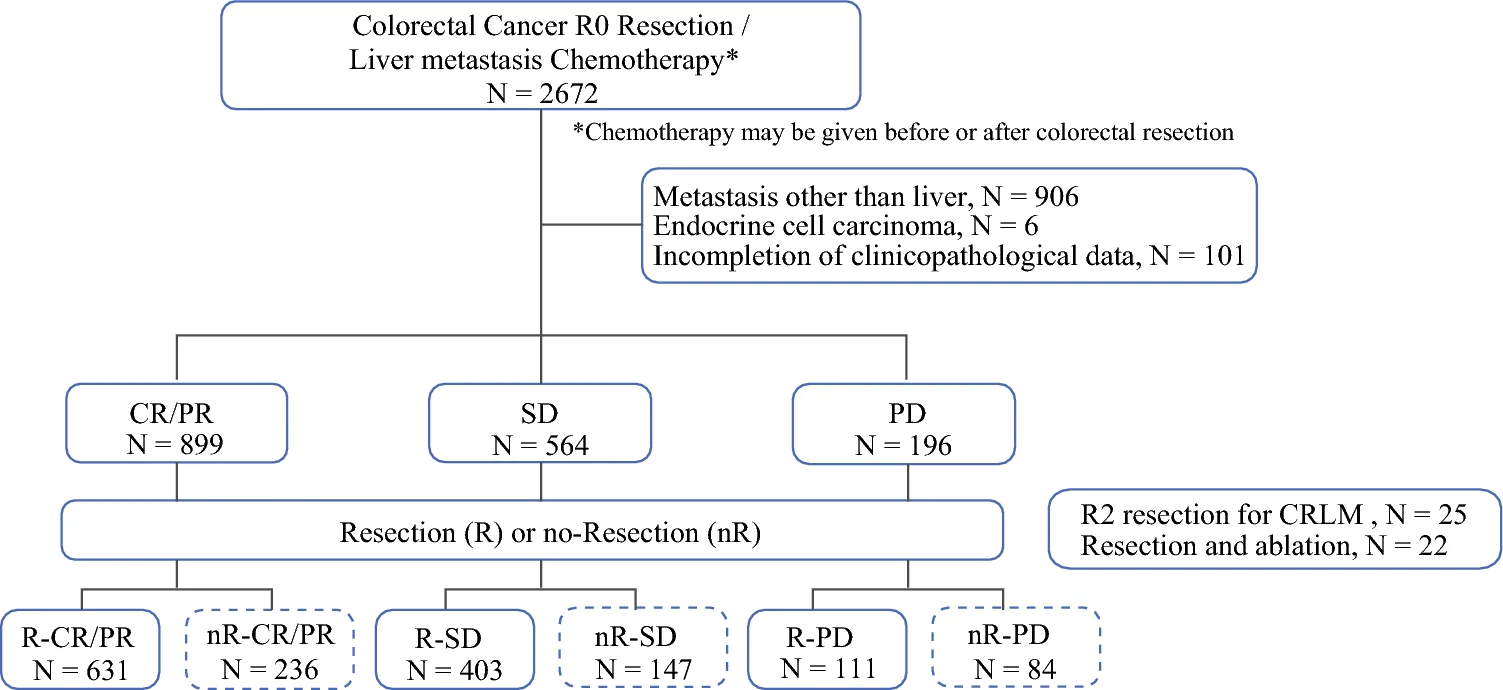
Flow diagram of patient selection and grouping. CR, complete response; CRLM, colorectal liver metastases; nR, no resection; PD, progressive disease; PR, partial response; R, resection; SD, stable disease

The database included 11,647 patients with CRLM. Of these, 2672 received preoperative chemotherapy for CRLM after R0 resection for colorectal cancer. As shown in Figure 1, a total of 1060 patients met the exclusion criteria, and the remaining 1612 patients were included in the final analysis. Of the remaining 8975 patients who were not included in the study population, 2489 had not undergone curative resection of the primary colorectal cancer, 5230 had received an initial treatment other than chemotherapy for CRLM, of whom 4648 underwent upfront surgery for CRLM, and 1256 had missing data for the inclusion criteria. Among the patients classified as having PD, it was not possible to determine whether the progression resulted from the appearance of new lesions or from an increase in the sum of the diameters of pre-existing lesions, because of database limitations. In the nR-PD group, two patients were evaluated as having PD because of the appearance of new extrahepatic metastases.

Table 1 presents the comparison of patient demographics between each treatment efficacy assessment. Regarding the factors of primary colorectal cancer, patients with PD were more likely to have pathologic node-positive colorectal cancers (vs CR/PR, *p*=0.009; vs SD, *p*=0.035). The interval from the diagnosis of CRLM to hepatectomy tended to be longer in the PD group (vs CR/PR, *p*=0.065; vs SD, *p*=0.002). Preoperative CEA levels in the PD group were significantly higher than those in the CR/PR and SD groups (vs CR/PR, *p*<0.001; vs SD, *p*<0.001). The proportion of patients who underwent hepatectomy was the lowest in the PD group (vs CR/PR, *p*<0.001; vs SD, *p*<0.001), and having five or more CRLMs was also the lowest (vs CR/PR, *p*<0.001 vs SD, *p*=0.006). Supplemental Table 1 lists the comparison of the demographics of patients who did and did not undergo resection, separately. The proportion of patients with four or fewer CRLMs was significantly higher in the R-PD group (87%) than in the nR-CR/PR (41%, *p*<0.001) and nR-PD (55%, *p*<0.001) groups.

**Table 1:** Patient characteristics

Characteristic	CR/PR (N=867)	SD (N=550)	PD (N=195)	CR/PR vs PD *p*-value	SD vs PD *p*-value
*Patient parameter*					
Sex					
Male	589 (68)	323 (59)	121 (62)	0.004^*^	0.416
Female	278 (32)	227 (41)	74 (38)		
Median age, y (25^th^; 75^th^)	66 (58; 73)	66 (60; 73)	67 (57; 73)	0.699	0.638
BMI, kg/m^2^ (25^th^; 75^th^)	22.3 (20.0; 24.9)	21.9 (19.6; 24.2)	21.6 (19.7; 24.3)	0.003^*^	0.727
HBs antigen					
Positive	12 (1)	14 (3)	2 (1)	1.000^a^	0.263^a^
Negative	809 (93)	508 (92)	182 (93)		
Unknown	46 (6)	28 (5)	11 (6)		
HCV antibody					
Positive	16 (2)	18 (3)	6 (3)	0.269^a^	1.000^a^
Negative	796 (92)	503 (91)	177 (91)		
Unknown	55 (6)	29 (6)	12 (6)		
*Colorectal cancer parameter*					
Location					
Colon	553 (64)	332 (60)	121(62)	0.198	0.645
Rectum	309 (35)	211 (38)	73 (37)		
Colon and rectum	5 (1)	7 (2)	1 (1)		
Adjuvant chemotherapy					
Yes	99 (11)	88 (16)	60 (31)	<0.001^* a^	0.004^* a^
No	99 (11)	51 (9)	13 (7)		
Unknown	669 (78)	411 (75)	122 (62)		
KRAS					
Mutant	160 (18)	134 (24)	50 (25)	<0.001^* a^	0.130 ^a^
Wild type	354 (41)	181 (33)	47 (24)		
Unknown	353 (41)	235 (43)	98 (51)		
pT stage ≥3	818 (94)	510 (93)	191 (98)	0.220	0.007^*^
pN stage ≥1	610 (70)	395 (72)	156 (80)	0.009^*^	0.035^*^
Histological differentiation					
Well or moderate	805 (93)	492 (89)	167 (86)	0.135	0.035^*^
Poorly	62 (7)	58 (11)	28 (14)		
Lymphatic invasion, yes	612 (71)	387 (70)	149 (76)	0.928	0.106
Vascular invasion, yes	711 (82)	435 (79)	157 (81)	0.173	0.672
Liver metastasis parameter					
Hepatectomy performed	631 (73)	403 (73)	111 (57)	< 0.001^*^	< 0.001^*^
CRLM diagnosis-hepatectomy interval, month (25^th^, 75^th^)	6.3 (5.0, 9.1)	6.1 (4.3; 8.5)	7.2 (5.3; 10.1)	0.065	0.002^*^
Prechemotherapy CEA, ng/mL (25^th^, 75^th^)	12.8 (4.2, 83.8)	15.8 (4.1; 117.6)	12.8 (4.2; 78)	0.366	0.265
Preoperative CEA, ng/mL (25^th^, 75^th^)	4.8 (2.7, 11.9)	7.6 (3.5; 36.2)	21.7 (6.2; 92.8)	< 0.001^*^	< 0.001^*^
Metastasis number ≥5	385 (44)	206 (37)	52 (27)	< 0.001^*^	0.006^*^
Maximum size >50 mm	214 (25)	146 (27)	41 (21)	0.698	0.149
Liver metastasis timing					
Synchronous	666 (77)	401 (73)	127 (65)	0.096	0.039^*^
Metachronous	201 (23)	149 (27)	68 (35)		
Hilar lymph nodes metastasis, yes	10 (1)	9 (2)	2 (1)	0.411	0.543
*Chemotherapy parameter*					
Regimen					
Including anti-EGFR agents	70 (8)	27 (5)	12 (6)	<0.001^*a^	0.005^*a^
Including anti-VEGF agents	339 (38)	221 (40)	55 (28)		
Other regimen	227 (26)	226 (41)	99 (51)		
Oxaliplatin-based regimen without molecular-targeted agents	158 (18)	134 (24)	47 (24)		
Irinotecan-based regimen without molecular-targeted agents	30 (3)	22 (4)	5 (3)		
Oral fluoropyrimidine/5-FU-based regimen	39 (5)	70 (13)	47 (24)		
Unknown	231 (28)	76 (14)	29 (15)		

Data are presented as n (%) unless otherwise indicated.

^*^ Statistically significant.

^a^ Unknown status was excluded from the analysis

*P*-values for regimen were calculated using the three major categories: anti-EGFR-containing, anti-VEGF-containing, and other regimens. Oxaliplatin-based regimens included FOLFOX, XELOX/CAPOX, SOX, and FOLFOXIRI without molecular-targeted agents. Irinotecan-based regimens included FOLFIRI, CPT-11-containing regimens, IRIS, IFL, and TEGAFIRI without molecular-targeted agents. Oral fluoropyrimidine/5-FU-based regimens included 5-FU/l-LV, UFT/LV, TS-1, capecitabine, and related fluoropyrimidine-based regimens.

BMI, body mass index; CEA, carcinoembryonic antigen; CR, complete response; CRLM, colorectal liver metastases; EGFR, epidermal growth factor receptor; HBs, hepatitis B, surface antibody; HCV, hepatitis C virus; PD, progressive disease; PR, partial response; SD, stable disease; VEGF, vascular endothelial growth factor; 5-FU, fluorouracil

### DFS, Cumulative Incidence of Recurrence, and Sites of Initial Recurrence

**Fig. 2:**
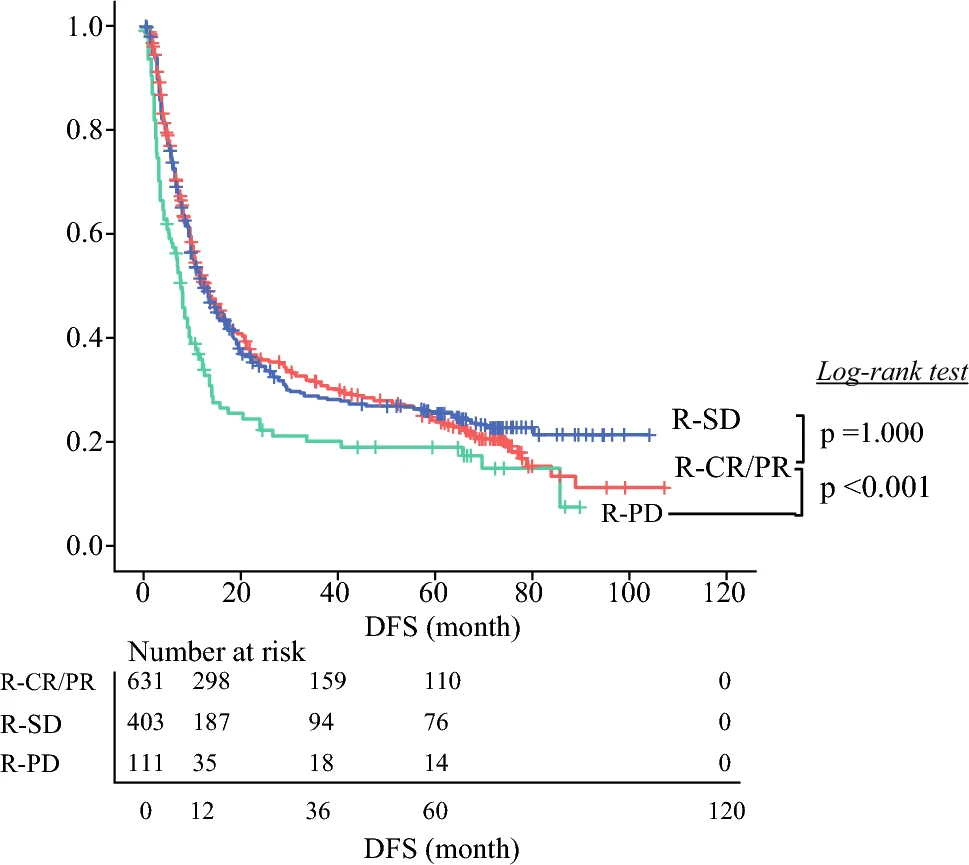
Disease-free survival (DFS) curves of patients according to the therapeutic effect after chemotherapy. CR, complete response; PD, progressive disease; PR, partial response; R, resection; SD, stable disease

The median DFS of the patients with R-CR/PR, R-SD, and R-PD was 12.6, 12.0, and 7.8 months, respectively (Figure 2). DFS rates at 1, 3, and 5 years in the R-PD group were 35.9%, 20.0%, and 18.9%, respectively, and were significantly worse than those of the CR/PR (51.6%, 31.0%, and 24.6%, respectively; *p*<0.001) and R-SD (49.9%, 28.4%, and 25.6%, respectively; *p*<0.001) groups.

The 6-month and 1-year cumulative incidence of recurrence differed significantly among the three groups (Gray’s test, *p*<0.001), with the highest incidence being observed in the R-PD group. The cumulative incidence of recurrence in patients with R-CR/PR, R-SD, and R-PD was 26.0% (95% confidence interval [CI] 22.6–29.6), 26.3% (95% CI 22.0–30.7), and 41.0% (95% CI 31.7–50.0), respectively, at 6 months after hepatectomy, and 46.9% (95% CI 42.8–50.8), 49.6% (95% CI 44.5–54.4), and 63.2% (95% CI 53.2–71.7), respectively, at 1 year after hepatectomy.

Table 2 presents a comparison of the incidence of recurrence and sites of initial recurrence between the patients with R-PD and those with R-CR/PR/SD. Among the patients with tumor recurrence limited to the liver, no significant differences existed in the site (local vs distant, *p*=0.685), number (*p*=0.498), or maximum size (*p*=0.274) of recurrent lesions. Conversely, the frequency of repeat hepatectomy was higher in the R-CR/PR/SD group.

**Table 2:** Recurrence status and treatment after relapse

Status and treatment		R-CR/PR/SD N = 1034	R-PD N = 111	*p*-Value
All data				
Any recurrence		695 (67)	87 (78)	0.017^*^
Recurrence location	Liver recurrence	326 (47)	42 (49)	0.331
	Extrahepatic recurrence	261 (38)	27 (31)	
	Liver and extrahepatic recurrence	108 (15)	18 (20)	
Liver-limited recurrence		N=326	N=42	
Liver recurrence sites	Local recurrence	70 (21)	7 (16)	0.685^a^
	Distant recurrence	252 (77)	34 (81)	
	Unknown	4 (1)	1 (2)	
Liver recurrence status	Recurrence number ≥5	49 (15)	8 (19)	0.498
	Maximum size >50 mm	7 (2)	2 (5)	0.274
Treatment after liver recurrence	Resection	151 (47)	9 (23)	0.018^*^
	Chemotherapy	106 (32)	20 (47)	
	Radiation	3 (1)	0 (0)	
	RFA/MCT	11 (3)	1 (2)	
	BSC	55 (17)	12 (28)	

Data are presented as n (%) unless otherwise indicated.

^*^ Statistically significant.

^a^ Unknown status was excluded from the analysis

BSC, best supportive care; MCT, microwave coagulation therapy; PD, progressive disease; R-CR, resection to complete response; RFA, radiofrequency ablation; R-PD, resection to progressive disease

### Survival Analyses

**Fig. 3:**
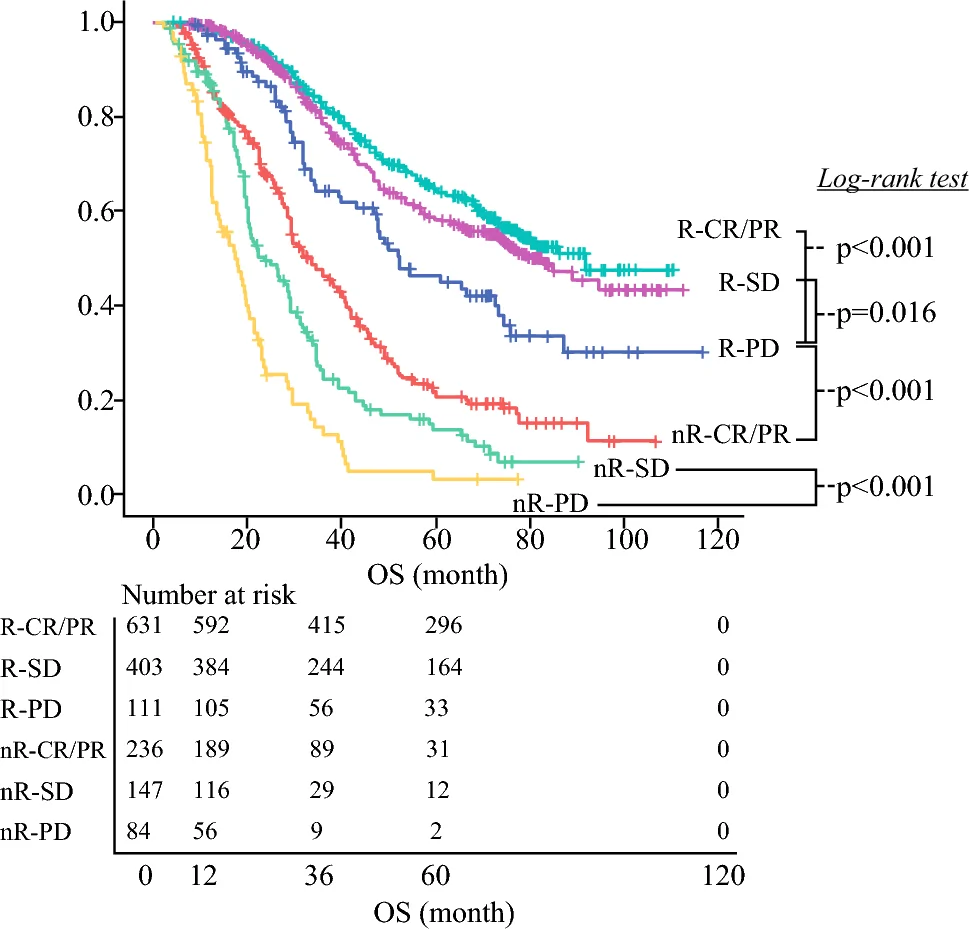
Kaplan–Meier curve for overall survival (OS) of patients according to the therapeutic effect after chemotherapy. CR, complete response; nR, no resection; PD, progressive disease; PR, partial response; R, resection; SD, stable disease

The Kaplan–Meier curves for OS from the date of CRLM diagnosis of six groups based on chemotherapy response and surgical resection status are shown in Figure 3. The median OS of patients with R-PD was 52.3 months, and their actuarial 1-, 3- and 5-year survival rates were 97.3%, 64.3%, and 46.5%, respectively. The median OS of nR-PD was 17.4 months, and their actuarial 1-, 3- and 5-year survival rates were 70.7%, 14.2%, and 3.2%, respectively. OS of the R-PD group was worse than that of the R-SD group (5 year, 57.9%, *p*=0.016) but was better than that of the nR-CR/PR group (5 year, 20.5%, *p*<0.001). OS of the nR-PD was significantly worse than that of the nR-SD group (5 year, 13.5%, *p*<0.001). Separate analysis of the R-CR and R-PR groups showed 5 year OS rates of 63.1% and 64.7%, respectively. Supplemental Figure 1 shows the Kaplan–Meier curves comparing OS among the patients with nR-CR, nR-PR, and R-PD. The actuarial 5-year survival rates of those with nR-CR and nR-PR were 47.1% and 11.4%, respectively. OS was comparable between the R-PD and nR-CR groups (*p*=1.000).

**Fig. 4:**
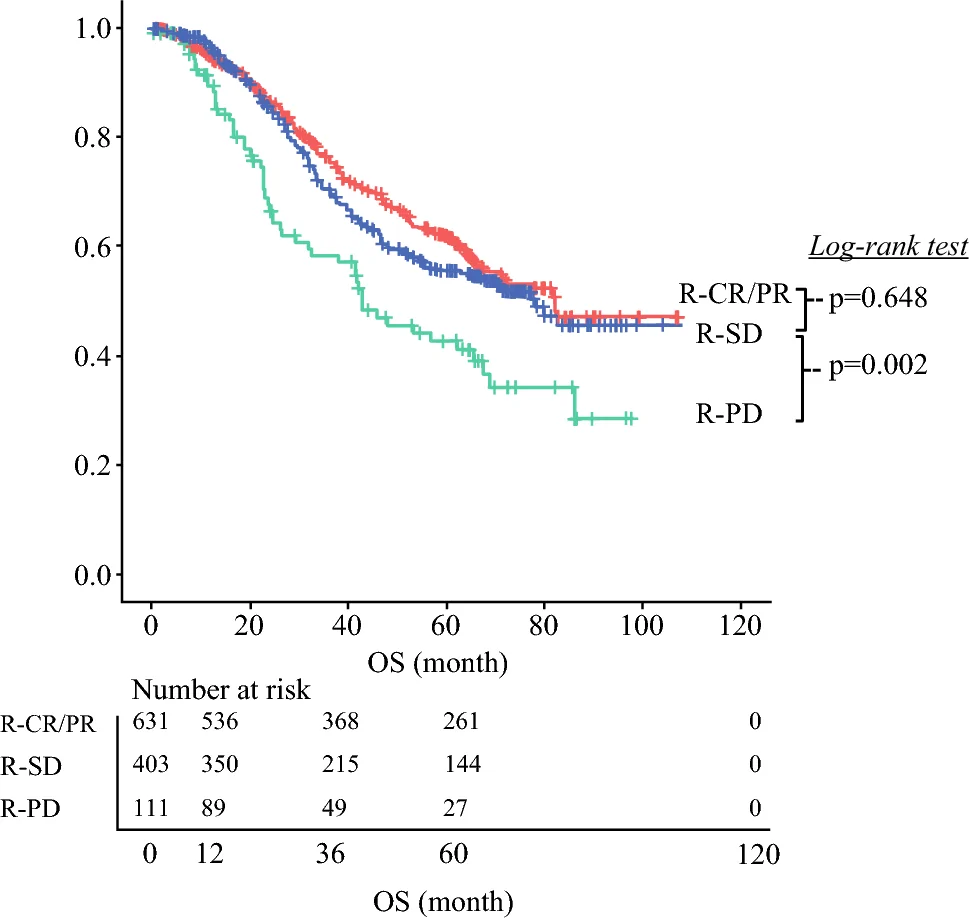
Kaplan–Meier curve for overall survival (OS) after hepatectomy. CR, complete response; nR, no resection; PD, progressive disease; PR, partial response; R, resection; SD, stable disease

Kaplan–Meier curve for postoperative OS from the time of hepatectomy is shown in Figure 4. The median OS of the patients with R-PD was 42.9 months, and their actuarial 1-, 3- and 5-year survival rates were 89.4%, 58.4%, and 42.7%, respectively. OS of the R-PD group was significantly worse than that of the R-CR/PR (median OS, 82.2 months; 5 year, 62.0%; *p*<0.001) and R-SD (median OS, 78.1 month; 5 year, 55.5%; *p*<0.001) groups.

The results of the multivariate analysis for predictors associated with poor survival following hepatectomyin the R-PD group are presented in Table 3. Multivariate analysis revealed that preoperative CEA ≥100 ng/mL and metachronous CRLM were independent predictors of worse OS. The corresponding E-values were 3.77 for the point estimate and 1.72 for the lower limit of the 95% CI for CEA ≥100 ng/ml, and 3.04 and 1.35, respectively, for metachronous CRLM. In the R-PD group, tumor characteristics, recurrence rates, recurrence sites, and treatments after recurrence were comparable between the patients with synchronous and metachronous CRLM except for the proportion in whom adjuvant chemotherapy was performed following primary colorectal resection (Supplemental Table 2). Among patients with metachronous CRLM who underwent hepatectomy, the median interval from primary colorectal surgery to CRLM diagnosis was 6.4, 8.5, and 7.3 months in the R-CR/PR, R-SD, and R-PD groups, respectively, and did not differ significantly among the three groups (*p*=0.076). The median OS after post-hepatectomy recurrence of metachronous and synchronous CRLM was 17.8 and 34.0 months, respectively (*p*=0.418). Conversely, among patients who underwent chemotherapy as post-recurrence treatment, the median OS after recurrence of metachronous and synchronous CRLM was 17.8 and 36.5 months, respectively, with significant difference (*p*=0.008).

**Table 3:** Univariate and multivariate analysis of risk factors for worse overall survival (OS) in the resection to progressive disease (R-PD) group

Characteristic	R-PD group N = 111				
	N (%)	Median OS (month)^a^	Univariate *p*-value	Multivariate	
				*p*-Value	HR (95% CI)
*Patient parameter*					
Sex					
Male	70 (63)	42.0	0.301		
Female	41 (37)	NR			
Age, years					
≥70	21 (19)	45.9	0.646		
<70	90 (81)	42.0			
BMI, kg/m^2^					
<18	6 (5)	22.8	0.137		
≥18	105 (95)	45.9			
*Colorectal cancer parameter*					
Location					
Rectum	40 (36)	38.0	0.602		
Colon	71 (64)	42.9			
Adjuvant chemotherapy					
No	7 (6)	55.4	0.282		
Yes	40 (36)	24.7			
KRAS					
Mutant	35 (32)	32.0	0.161		
Wild	26 (23)	53.4			
pT stage					
≥3	107 (96)	42.9	0.778		
≤2	4 (4)	22.8			
pN stage					
≥1	86 (77)	45.9	0.359		
0	25 (23)	24.7			
Differentiation					
Poorly	8 (7)	NR	0.525		
Well or moderate	103 (93)	42.9			
*Liver metastasis parameter*					
CRLM diagnosis-hepatectomy interval					
>6 months	69 (62)	42.0	0.480		
≤6 months	42 (38)	53.4			
Prechemotherapy CEA					
≥100 ng/mL	17 (15)	20.1	0.143		
<100 ng/mL	94 (85)	42.9			
Preoperative CEA					
≥100 ng/mL	25 (23)	20.3	0.014^*^	0.009^*^	2.17 (1.208–3.923)
<100 ng/mL	86 (77)	42.9			
Metastasis number					
≥5	14 (13)	NR	0.742		
≤4	97 (87)	42.9			
Maximum size					
>50 mm	26 (23)	32.0	0.406		
≤50 mm	85 (77)	45.9			
Liver metastasis timing					
Metachronous	44 (40)	24.7	0.039^*^	0.026^*^	1.82 (1.073–3.088)
Synchronous	67 (60)	56.9			
Hilar lymph nodes metastasis					
Yes	1 (1)	22.3	0.215		
No	110 (99)	42.9			
*Chemotherapy parameter*					
Only first-line chemotherapy before hepatectomy					
No	22 (20)	41.5	0.701		
Yes	89 (80)	45.9			
Regimen					
Anti-EGFR agents	5 (4)	NR	0.261		
Anti-VEGF agents	34 (31)	42.9			
Other	72 (65)	41.5			

^*^ Statistically significant.

^a^ OS was calculated from hepatic resection.

BMI, body mass index; CEA, carcinoembryonic antigen; CI, confidence interval; CRLM, colorectal liver metastases; EGFR, epidermal growth factor receptor; HR, hazard ratio; NR, not reached; VEGF, vascular endothelial growth factor

## Discussion

We utilized a Japanese nationwide database to focus on patients who exhibited PD after systemic chemotherapy for CRLM. Furthermore, we evaluated their survival outcomes and prognostic factors following hepatectomy. We found that 12% of patients were assessed as PD after chemotherapy; among them, 57% underwent hepatectomy, significantly lower than the numbers in the CR/PR and SD groups. Further, although a potential selection bias existed in patient background, OS was significantly better in the R-PD group than in the nR-PR, nR-SD, and nR-PD groups. Lastly, in the R-PD group, preoperative CEA ≥100 ng/mL and metachronous CRLM were independent predictors of poor OS. These findings suggest that, even in patients with PD, hepatectomy may offer a survival benefit; however, careful patient selection based on chemotherapy response and clinical background is essential when determining surgical indications.

In general, PD has been regarded as a predictor of poor prognosis, and patients with tumor progression during chemotherapy have usually been deemed unsuitable for surgical resection.^16–18^ However, previous studies have not thoroughly investigated whether PD itself should be an absolute contraindication for hepatectomy.^8,18^ Here, hepatectomy aimed at improving the prognosis of patients in the PD group also appears to be a supportive strategy in selected cases. The 5 year postoperative OS of 42.7% appeared superior to that of patients who achieved conversion hepatectomy for initially unresectable disease, reported as 8–35%.^8,18,19^ However, the potential influence of selection bias should not be ignored when interpreting the outcomes of survival analysis. Patients in the R-PD group were younger and had a significantly lower proportion of cases with five or more tumors than those in the nR-CR/PR and nR-SD groups. Although the Joint Committee of Liver Metastases Survey from Colorectal Cancer did not define explicit criteria for hepatectomy, the presence of multiple tumors is definitely a poor prognostic factor.^19–21^ The apparent survival advantage of the R-PD group over the nR-PR group may also have been influenced by unresectability bias and lead-time-related or time-dependent selection bias because the non-resected groups may have included more patients with oncologically or technically unresectable disease irrespective of radiological response to chemotherapy, whereas patients in the R-PD group were selected for hepatectomy only if they maintained adequate performance status and resectability despite radiological progression. Therefore, the favorable long-term outcomes observed in the R-PD group compared with the non-resected groups, including a postoperative 5-year OS of 42.7%, should not be extrapolated to the broader population of patients with PD. Rather, this survival outcome should be interpreted as one achieved in a highly selected group of patients who had adequate performance status and relatively limited tumor burden and in whom hepatectomy was considered technically feasible and oncologically meaningful by experienced surgical teams. The database also did not allow further stratification of PD according to the tempo or pattern of progression, such as slow versus rapid progression, which may have influenced both surgical decision-making and postoperative outcomes. Conversely, objective response to preoperative chemotherapy is generally considered to be associated with better oncologic outcomes; however, postoperative prognosis was comparable between the R-CR/PR and R-SD groups in the present study. One possible explanation is that RECIST-based radiological response may not fully capture clinically relevant changes in CRLM after chemotherapy, such as morphologic response on imaging and tumor marker kinetics.^22^

The significantly longer interval from CRLM diagnosis to hepatectomy in the PD group may partly reflect both prior adjuvant chemotherapy for primary colorectal cancer and the clinical evaluation process before hepatectomy for CRLM. Adjuvant chemotherapy after primary colorectal resection was recorded in 36% (40/111) of the R-PD group compared with 12% (78/631) of the R-CR/PR group and 19% (76/403) of the R-SD group, although unknown status was the most prevalent. Such prior chemotherapy exposure may have influenced subsequent treatment response or liver condition, and in some patients, additional time may have been required to allow hepatic recovery before surgery. However, when focusing specifically on chemotherapy for CRLM, the longer interval was unlikely to be explained solely by multiple treatment lines before hepatectomy because 89 of 111 patients (80%) in the R-PD group underwent hepatectomy after first-line chemotherapy for CRLM. Thus, the prolonged interval from CRLM diagnosis to hepatectomy in the R-PD group, as presented in Table 1, may also reflect the time required to evaluate treatment response, confirm the tempo of progression, and determine whether hepatectomy remained feasible.

In Japan, preoperative chemotherapy is not routinely recommended for technically resectable CRLM according to the Japanese Society for Cancer of the Colon and Rectum guidelines 2024 for colorectal cancer treatment.^23^ Although the definition of resectability remains unclear, the European Organization for Research and Treatment of Cancer defines resectable CRLM as technically resectable and lesions with up to four metastases.^24^ The PD group likely included a substantial number of patients with features increasingly recognized as “borderline resectable” or “not optimal for resection” for reasons such as high tumor burden, large tumor size, vascular invasion, or elevated tumor markers.^24,25^ Although elevated tumor markers may have contributed to treatment selection, the median prechemotherapy CEA values as presented in Table 1 were relatively modest. However, this should be interpreted with caution because CEA levels showed substantial variety within each group, and prechemotherapy CEA tended to be higher in the non-resected subgroups (Supplementary Table 1). These findings suggest that the patients with higher CEA were less likely to proceed to hepatectomy. These tumor-related factors, along with the biologic factor of disease progression, may explain the significantly higher recurrence rates within 1 year following hepatectomy in the R-PD group. The significantly lower rate of repeat hepatectomy in this group, despite the size and number of recurrent tumors being comparable to those in the CR/PR and SD groups, may reflect concerns regarding a short disease-free interval from the initial hepatectomy or limited surgical tolerance due to insufficient remnant liver function. Therefore, PD status itself should warrant careful consideration for the indication of hepatectomy. Ideally, patients undergoing preoperative chemotherapy with technically resectable CRLM should be distinguished from those undergoing conversion therapy for initially ‘unresectable’ disease.^26^ However, in the absence of an objective and universally accepted definition of ‘unresectable CRLM’ or ‘oncologically optimal CRLM for resection’, making a clear distinction between these clinical scenarios remains challenging. The present results should be interpreted alongside recognition that all patients who received preoperative chemotherapy were included without considering initial resectability. Molecular tumor characteristics should also be considered when interpreting the present findings. KRAS status is clinically relevant not only for selecting anti-EGFR therapy but also as a prognostic biomarker in patients undergoing hepatectomy for CRLM. Previous studies have reported that KRAS mutation has been associated with worse survival and distinct recurrence patterns after resection of CRLM.^9,10^ However, in the present study, KRAS status was unknown in 51% of patients with PD, limiting our ability to fully assess the influence of KRAS status and KRAS-guided molecular-targeted therapy on postoperative survival. Therefore, although KRAS mutation was not revealed as a prognostic predictor in the R-PD group, this finding should be interpreted cautiously. More broadly, RECIST-based radiological response reflects changes in tumor size but does not fully capture the biological aggressiveness of CRLM. Future assessment of disease behavior and surgical candidacy may therefore require integration of radiological response with molecular profiling, including RAS/BRAF status and potentially circulating tumor DNA.^27–29^ such an approach may help identify patients with radiological progression who nevertheless have surgically controllable disease, as well as patients with radiological response who remain poor candidates for hepatectomy because of aggressive tumor biology. Thus, molecular profiling may refine RECIST-based response assessment and improve the selection of optimal candidates for hepatectomy.

In the analysis of the R-PD group, metachronous CRLM emerged as an independent prognostic factor. The result seems paradoxical because synchronous liver metastasis is widely recognized as a poor prognostic factor.^30,31^ The E-value analysis suggested that the observed association between metachronous CRLM and worse prognosis was moderately robust to unmeasured confounding. An unmeasured confounder would need to be associated with both metachronous CRLM and worse prognosis by a risk ratio of approximately 3.0 each to fully explain away the observed hazard ratio of 1.82, whereas an association of 1.35 each would be sufficient to shift the lower limit of the 95% CI to the null. This finding suggested that the association was weakly significant. Although the significance showed limited robustness, the emergence of metachronous CRLM as a poor prognostic factor may be attributable to chemoresistance. In the present study, the interval from primary colorectal surgery to CRLM diagnosis among patients with metachronous CRLM was not significantly shorter in the R-PD group than in the R-CR/PR or R-SD groups. At metachronous CRLM diagnosis, oxaliplatin-based adjuvant chemotherapy for primary colorectal cancer was completed over 3–6 months per standard treatment especially in patients with stage III disease.^32–34^ Although the exact number of patients who received adjuvant chemotherapy could not be determined because of missing data (Table 1), prior adjuvant chemotherapy for primary colorectal cancer may have contributed to greater cumulative exposure to systemic therapy before hepatectomy in patients with metachronous CRLM than in those with synchronous metastases. Typically, systemic chemotherapy for metachronous CRLM is selected based on the number and size of the liver tumor as well as molecular profile of the primary colorectal tumor. The status of PD despite such treatment may serve as evidence that the tumor has undergone molecular evolution, acquiring additional somatic mutations and/or clonal selection of treatment-resistant subpopulations, possibly because of multiple prior lines of chemotherapy.^35–37^ In these cases, resistance to further systemic therapy for recurrence after hepatectomy could be anticipated. Consistent with this hypothesis, our study also demonstrated that the patients with metachronous CRLM showed significantly worse post-recurrence survival than those with synchronous metastases among those treated with chemotherapy for the recurrence. Therefore, surgical indications in patients with PD following chemotherapy for metachronous CRLM should be determined with caution, considering tumor status and the potential limited efficacy of further chemotherapy for future postoperative recurrence.

This study had some limitations. First, its retrospective design inherently introduces the potential for selection bias, as treatment decisions and patient inclusion were not randomized or controlled. Second, a considerable number of cases were excluded because data were missing, which may have led to selection bias, potentially affecting the robustness of the statistical analyses. Additionally, the database did not distinguish whether PD was due to the emergence of new lesions or enlargement of existing ones. Nor did it permit stratification of PD into slow versus rapid progression as mentioned. These differences may have important prognostic implications and have influenced the decision-making around proceeding to hepatectomy. Furthermore, among the non-resected patients, some were considered to have remained technically resectable, even after chemotherapy. The indications for hepatectomy were determined according to each institution’s criteria, which would include not only RECIST but also transition of tumor markers or progression-free interval. This information was not available in the database, so we could not determine why hepatectomy was or was not performed in each patient. This limitation should be taken into account when interpreting the survival outcomes of the non-resected groups. Third, many cases lacked information regarding KRAS status, the history of previous chemotherapy, including the adjuvant chemotherapy following colorectal surgery, and duration of chemotherapy; therefore, molecular and treatment-related confounding could not be fully addressed. The prognostic impact of adjuvant therapy following colorectal cancer resection is likely to be an important factor even in the R-PD group.^38^ However, preoperative chemotherapy regimens before hepatectomy were accurately documented, allowing for supplementary interpretation. More precise data regarding the previous treatment history could have shown other predictive factors affecting the survival outcomes of patients with PD.

In conclusion, PD after chemotherapy for CRLM should not be an absolute contraindication for hepatectomy, as postoperative 5-year OS of 42.7% was achieved in selected patients. Preoperative CEA ≥100 ng/mL and metachronous CRLM were identified as independent predictors of poor prognosis in patients with PD. Metachronous CRLM in the setting of PD after chemotherapy may reflect chemoresistant tumor biology after prior systemic therapy, including adjuvant chemotherapy for primary colorectal cancer, and should therefore be carefully considered when determining the indication for hepatectomy.

## Supplementary Information

Below is the link to the electronic supplementary material.

Supplementary file1 (JPEG 52 KB)

Supplementary file2 (DOCX 29 KB)

Supplementary file3 (DOCX 23 KB)

## Data Availability

The datasets generated and/or analyzed during the current study are not publicly available for ethical and legal restrictions. Researchers wishing to access the data may submit a request to the Joint Committee of Liver Metastases Survey from Colorectal Cancer, operated by the Japanese Society for Cancer of the Colon and Rectum and Japanese Society of Hepato-Biliary-Pancreatic Surgery. Data access will be granted upon review and approval by the committee, in accordance with the societies’ data-sharing policies and ethical guidelines.
