# Supplementary material for: Hepatectomy After Progressive Disease Following Chemotherapy for Colorectal Liver Metastases: A Japanese Nationwide Cohort Study
Source: Ann Surg Oncol. 2026 Aug 1;33(12):10001–12. doi: 10.1245/s10434-026-20283-2 (PMC13675336; doi:10.1245/s10434-026-20283-2)
Supplement: Supplementary file 1 [file 10434_2026_20283_MOESM2_ESM.docx]

Supp Table 1. Patient Characteristics Stratified by Chemotherapy Response and Hepatic Resection Status

|  | | R-CR/PR  (N=631) | R-SD  (N=403) | R-PD  (N=111) | nR-CR/PR  (N=236) | nR-SD  (N=147) | nR-PD  (N=84) | R-PD vs nR-CR/PR  P value | R-PD vs nR-PD  P value |
| --- | --- | --- | --- | --- | --- | --- | --- | --- | --- |
| Sex, n (%) | Male | 416 (66) | 235 (58) | 70 (63) | 173 (73) | 88 (60) | 51 (61) | 0.052 | 0.737 |
|  | Female | 215 (34) | 168 (42) | 41 (37) | 63 (27) | 59 (40) | 33 (39) |  |  |
| Median Age, y (25th, 75th) | | 65 (58, 73) | 66 (60, 73) | 62 (55, 72) | 67 (62, 74) | 66 (59, 74) | 69 (64, 75) | <0.001^†^ | <0.001^†^ |
| Location, n (%) | Colon | 398 (63) | 238 (59) | 71 (64) | 155 (66) | 94 (64) | 50 (60) | 0.739 | 0.447 |
|  | Rectum | 229 (36) | 158 (39) | 40 (36) | 80 (33) | 53 (36) | 33 (39) |  |  |
|  | Colon and Rectum | 4 (1) | 7 (2) | 0 (0) | 1 (1) | 0 (0) | 1 (1) |  |  |
| Adjuvant chemotherapy for colorectal cancer | Yes | 78 (12) | 76 (19) | 40 (36) | 8 (3) | 12 (8) | 20 (24) | <0.001^†^* | 0.381* |
|  | No | 69 (11) | 33 (8) | 7 (6) | 5 (2) | 18 (12) | 6 (7) |  |  |
|  | Unknown | 484 (77) | 294 (73) | 64 (58) | 223 (95) | 117 (80) | 58 (69) |  |  |
| KRAS, n (%) | Mutant | 123 (19) | 99 (25) | 35 (32) | 37 (16) | 35 (24) | 15 (18) | <0.001^†^* | 0.085* |
|  | Wild -type | 263 (42) | 137 (34) | 26 (23) | 91 (39) | 44 (30) | 21 (25) |  |  |
|  | Unknown | 245 (39) | 167 (41) | 50 (45) | 108 (45) | 68 (46) | 48 (57) |  |  |
| Colorectal cancer pT stage ≥3, n (%) | | 592 (94) | 367 (91) | 107 (96) | 226 (96) | 143 (97) | 84 (100) | 0.779 | 0.078 |
| Colorectal cancer pN stage ≥1, n (%) | | 441 (64) | 281 (70) | 86 (78) | 169 (49) | 114 (71) | 70 (81) | 0.327 | 0.311 |
| Colorectal cancer histological differentiation, n (%) | Well or moderate | 590 (94) | 358 (89) | 99 (89) | 215 (91) | 134 (91) | 68 (81) | 0.838 | 0.016^†^ |
|  | Poorly | 41 (6) | 45 (11) | 12 (11) | 21 (9) | 13 (9) | 16 (19) |  |  |
| Prechemotherapy CEA, ng/ml (25th, 75th) | | 10.9 (4.1, 61.9) | 11.7 (3.7, 69.8) | 9.6 (3.7, 47.8) | 24.5 (4.7, 240) | 53.8 (7, 255) | 17.1 (4.8, 110) | 0.001^†^ | 0.051 |
| Preoperative CEA, ng/ml (25th, 75th) | | 4.8 (2.7, 11.9) | 7.6 (3.5, 36.2) | 21.7 (6.2, 92.8) | - | - | - | - | - |
| CRLM number ≥5, n (%) | | 245 (39) | 105 (26) | 14 (13) | 140 (59) | 101 (69) | 38 (45) | <0.001^†^ | <0.001^†^ |
| CRLM maximum size, >50mm, n (%) | | 149 (24) | 84 (21) | 26 (23) | 65 (28) | 62 (42) | 15 (18) | 0.436 | 0.379 |
| Liver metastasis timing, n (%) | synchronous | 482 (76) | 275 (68) | 67 (60) | 184 (78) | 21 (14) | 60 (71) | <0.001^†^ | 0.108 |
|  | metachronous | 149 (24) | 128 (32) | 44 (40) | 52 (22) | 126 (86) | 24 (29) |  |  |
| Hilar lymph nodes metastasis, yes, n(%) | | 6 (1) | 2 (1) | 1 (1) | 4 (3) | 7 (4) | 1 (1) | 0.562 | 0.842 |
| Only first-line chemotherapy before hepatectomy, yes, n (%) | | 558 (88) | 336 (83) | 89 (80) | - | - | - | - | - |
| Chemotherapy for CRLM | Including anti-EGFR agents | 57 (9) | 23 (6) | 5 (4) | 13 (6) | 4 (3) | 7 (8) | 0.889 | 0.427 |
|  | Including anti-VEGF agents | 264 (42) | 162 (40) | 34 (31) | 75 (32) | 59 (40) | 21 (25) |  |  |
|  | Other regimen | 310 (49) | 218 (54) | 72 (65) | 148 (62) | 84 (57) | 56 (67) |  |  |

^†^ Statistically significant. * Unknown status was excluded from the analysis

CR, complete response; PR, partial response; SD, stable disease; PD, progressive disease; CRLM, colorectal liver metastasis; EGFR, epidermal growth factor receptor; VEGF, vascular endothelial growth factor
