# Supplementary material for: Hepatectomy After Progressive Disease Following Chemotherapy for Colorectal Liver Metastases: A Japanese Nationwide Cohort Study
Source: Ann Surg Oncol. 2026 Aug 1;33(12):10001–12. doi: 10.1245/s10434-026-20283-2 (PMC13675336; doi:10.1245/s10434-026-20283-2)
Supplement: Supplementary file 2 [file 10434_2026_20283_MOESM3_ESM.docx]

Supp Table 2. Comparison of characteristics according to the metastasis timing of R-PD group

|  | | Synchronous  (N=67) | Metachronous  (N=44) | P value |
| --- | --- | --- | --- | --- |
| Age ≥70 y | | 11 (18) | 10 (24) | 0.436 |
| Adjuvant chemotherapy for colorectal cancer, yes | | 3 (4) | 37 (84) | <0.001^†^ |
| CRLM diagnosis-hepatectomy interval > 6 month | | 45 (67) | 24 (54) | 0.180 |
| Prechemotherapy CEA >100 ng/ml | | 12 (18) | 5 (11) | 0.348 |
| Preoperative CEA >100 ng/ml | | 16 (24) | 9 (20) | 0.672 |
| Metastasis number ≥5 | | 10 (15) | 4 (9) | 0.365 |
| Maximum size>50mm | | 17 (25) | 9 (20) | 0.549 |
| Only first-line chemotherapy for CRLM before hepatectomy | | 54 (80) | 35 (79) | 0.891 |
| Preoperative chemotherapy regimen | Anti-EGFR agents | 3 (4) | 2 (4) | 1.000 |
|  | Anti-VEGF agents | 21 (31) | 13 (30) |  |
|  | Other | 43 (65) | 29 (66) |  |
| Recurrence, yes | | 51 (76) | 36 (82) | 0.638 |
| Recurrence location | Liver recurrence | 23 (45) | 19 (53) | 0.747 |
|  | Extrahepatic recurrence | 16 (31) | 11 (31) |  |
|  | Liver and extrahepatic recurrence | 12 (24) | 6 (17) |  |
| Liver recurrence status | Recurrence number ≥5 | 1 (4) | 4 (21) | 0.158 |
|  | Maximum size >50mm | 1 (4) | 1 (5) | 1.000 |
| Treatment after recurrence | Resection | 5 (10) | 6 (17) | 0.695 |
|  | RFA/MCT | 1 (2) | 0 (0) |  |
|  | Chemotherapy | 32 (63) | 22 (61) |  |
|  | Chemoradiation | 2 (4) | 0 (0) |  |
|  | BSC | 11 (21) | 8 (21) |  |

^†^ Statistically significant.

CRLM, colorectal liver metastasis; CEA, Carcinoembryonic Antigen; EGFR, epidermal growth factor receptor; VEGF, vascular endothelial growth factor; RFA, radiofrequency ablation; MCT, microwave coagulation therapy; BSC, best supportive care
